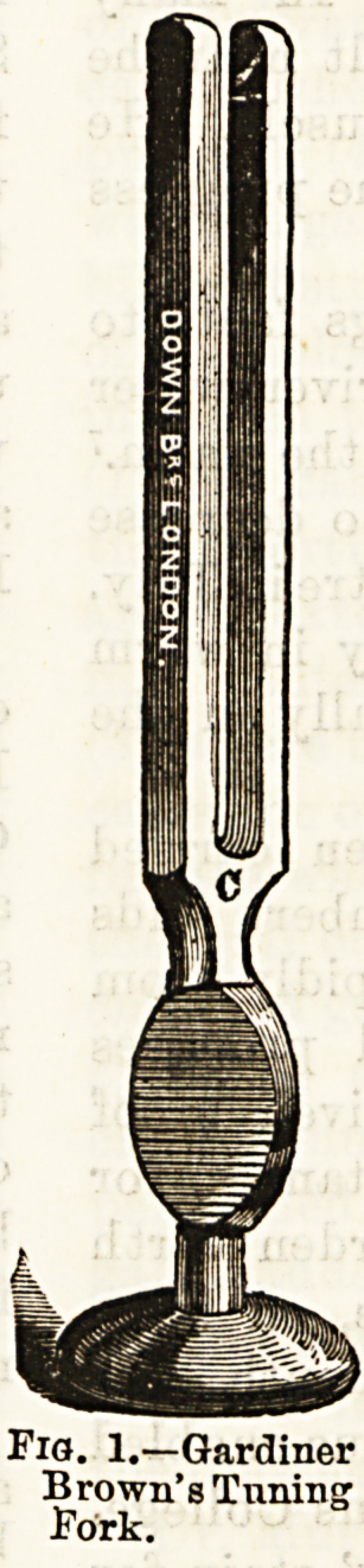# Diseases of the Ear

**Published:** 1894-06-30

**Authors:** P. Macleod Yearsley

**Affiliations:** Aural Surgeon and Surgeon of Farringdon General Dispensary, Assistant Demonstrator of Anatomy and Curator of Museum, and formerly Aural Clinical Assistant to Westminster Hospital


### DISEASES OF THE EAR.

By P. Macleod Yearsley, F.R.C.S.Eng., Aural Surgeon and Surgeon of Farringdon General Dispensary, Assistant Demonstrator of Anatomy and Curator of Museum, and formerly Aural Clinical Assistant to Westminster Hospital.

### I.—The Examination of the Ear.

From the frequency of errors in the diagnosis of diseases of the ear and the consequent want of success in their treatment, it is evident that an examination of the organ of hearing to ascertain the cause of deafness, pain, or tinnitus aurium is not so easy as it appears. I propose, therefore, to devote the first two articles of this series to the subject of physical examination, and although the necessary limits of space will not allow of an exhaustive discussion, I hope to at least indicate the essential points with which practitioners should be acquainted, and which they should on no occasion neglect. The first of all rules for the aurist is *routine*. If we are to do the best for our patients we shall more certainly attain our end by a systematic method of examination—that is to say, not only should the history (present, previous, and family) be carefully inquired into, the ear itself examined, but the nose, fauces, and naso-pharynx, as well as the general constitution, should be searched to find a cause for the disease. Even in cases where the root of the evil is quickly and easily discovered, it is as well to continue the examination systematically, as then one cannot reproach oneself for any after-complication that may arise. True, an exhaustive inquiry takes up a great deal of time, and is therefore often impossible to a busy practitioner, but were it done in every case there would be fewer patients with hearing organs ruined for the want of careful examination and timely appropriate treatment. It matters but little how the routine examination is done, provided that it is done, and every man will have his own order of its execution.

The next essential point is *gentleness*; rough manipulation may do very serious harm, and here it is well to bear in mind that certain methods not only help to inform us as to the state of the ear, but act to a greater or less exent as therapeutic agents, such for instance as the eustachian catheter and Politzer's apparatus. Further, it cannot be too often insisted that no manipulations, either for purposes of examination or treatment, should be carried out upon the ear without perfect illumination, and, unless this be at hand, it is better to leave the case alone than to grope blindly in imperfect light.

In taking the history of an aural case, questions must be asked regarding hereditary tendency, previous illness, diathesis, &c., since the answers elicited will often result in valuable information. The occurrence of any of the exanthemata should not only be ascertained, but the severity of the attack, its mode of onset, the age at which it occurred, and its sequelæ should be the subject of special questions. The history once obtained, the practitioner should before commencing any physical examination, proceed to make himself acquainted with the presence or absence of the four cardinal symptoms of ear disease—pain, deafness, discharge, and tinnitis. The indications of these signs will be gathered as these articles progress, but it may now be pointed out that the following special inquiries should be made in every case.

*Pain.*—Character, locality, duration, mode of onset, time when worse, severity, and relation to other symptoms.

*Deafness.*—Character, duration, mode of onset, degree, persistence, when better or worse, relation to changes in weather or surroundings, and to other symptoms.

*Discharge.*—Character, duration, mode of onset, amount, colour, odour, consistence, the presence of blood, whether preceded by pain, relation to changes in weather or surroundings, and to other symptoms.

*Tinnitus.*—Duration, when better or worse, degree, persistence, relation to weather or surroundings and to other symptoms, and above all, the character of the noises complained of.

Having obtained such information as can be gained by questioning, the hearing should be tested with the watch and tuning-fork. The former, although not a very delicate test, will be quite sufficient for an ordinary case, and is, moreover, most convenient in general practice. As different watches vary in the loudness of their ticking, the surgeon should test his own upon a person whose ears are known to be healthy, and having gained a knowledge of the normal hearing distance of that watch, should always use it for testing purposes. In testing the hearing power, take the best ear first, the other being closed by the patient's finger; the watch should be placed some way from the ear, and gradually brought nearer until the distance at which its ticking can be faintly heard is arrived at; this should be noted, and the other ear similarly examined. When the watch test has been applied, note should be made of the condition of the hearing as regards conversation, and the two tests compared. This is especially important, as some patients that can scarcely hear the watch can carry on conversation in the ordinary tone with ease, and *vice versâ.* Here it may be said that with most deaf persons there is no need whatever for shouting. A clear tone of ordinary loudness, provided the words are distinctly pronounced and not too quickly spoken, will often be far more successful than the painful shouting which many people seem to think is necessary.

Fig. 1.—Gardiner Brown's Tuning Fork.

The tuning-fork is an instrument which gives valuable information, enabling us to distinguish between disease of the middle and internal ear; its guidance is, however, not always unfailing. A good tuning-fork is that suggested by Gardiner Brown, of which an illustration is given (Fig. 1). It has, between the foot and fork, a roughened plate, which, when held between the finger and thumb, communicates to them the vibrations of the fork, so giving a simple and accurate standard for determining the condition of the auditory nerve.

The ear consisting of two essential parts, sound conducting and sound perceiving, it is of great importance to distinguish which is affected by disease. For this the vibrating tuning-fork should be placed on the patient's forehead, when, if he should be suffering from disease of the sound-conducting portion, he will hear the note best in the deaf or worst ear, the sound being intensified by the obstruction caused by the disease. One suffering with nerve disease will, on the contrary, hear the note less clearly with the diseased side. Further, in normal persons, when the tuning-fork can no longer be heard on the mastoid process, it is audible when held near the meatus, a fact which does not hold good in uncomplicated middle ear affections. The examination with the tuning-fork, to be again considered later, is one which should be repeated until the aurist is sure of its results, for true auditory nerve disease is less common than is supposed.

## Figures and Tables

**Fig. 1. f1:**